# The Effect of Endurance Training on Brain-Derived Neurotrophic Factor and Inflammatory Markers in Healthy People and Parkinson's Disease. A Narrative Review

**DOI:** 10.3389/fphys.2020.578981

**Published:** 2020-11-19

**Authors:** Paulina Małczyńska-Sims, Małgorzata Chalimoniuk, Anna Sułek

**Affiliations:** ^1^Department of Genetics, Institute of Psychiatry and Neurology, Warsaw, Poland; ^2^Department of Physical Education and Health in Biała Podlaska, Józef Piłsudski University of Physical Education in Warsaw, Warsaw, Poland

**Keywords:** BDNF, Parkinson's disease, endurance training, inflammation, brain-derived neurotrophic factor, healthy young adults, healthy older adults

## Abstract

**Background:** One purpose of the training conducted by people is to lose bodyweight and improve their physical condition. It is well-known that endurance training provides many positive changes in the body, not only those associated with current beauty standards. It also promotes biochemical changes such as a decreased inflammatory status, memory improvements through increased brain-derived neurotrophic factor levels, and reduced stress hormone levels. The positive effects of training may provide a novel solution for people with Parkinson's disease, as a way to reduce the inflammatory status and decrease neurodegeneration through stimulation of neuroplasticity and improved motor conditions.

**Aim:** This narrative review aims to focus on the relationship between an acute bout of endurance exercise, endurance training (continuous and interval), brain-derived neurotrophic factor and inflammatory status in the three subject groups (young adults, older adult, and patients with Parkinson's disease), and to review the current state of knowledge about the possible causes of the differences in brain-derived neurotrophic factor and inflammatory status response to a bout of endurance exercise and endurance training. Furthermore, short practical recommendations for PD patients were formulated for improving the efficacy of the training process during rehabilitation.

**Methods:** A narrative review was performed following an electronic search of the database PubMed/Medline and Web of Science for English-language articles between January 2010 and January 2020.

**Results:** Analysis of the available publications with partial results revealed (1) a possible connection between the brain-derived neurotrophic factor level and inflammatory status, and (2) a more beneficial influence of endurance training compared with acute bouts of endurance exercise.

**Conclusion:** Despite the lack of direct evidence, the results from studies show that endurance training may have a positive effect on inflammatory status and brain-derived neurotrophic factor levels. Introducing endurance training as part of the rehabilitation in Parkinson's disease might provide benefits for patients in addition to pharmacological therapy supplementation.

## Introduction

Parkinson's disease (PD) is one of the most common neurodegenerative diseases in the population of people over 60 years of age (Lew, 2007). Despite the systematic improvement of diagnosis and discovery of new possible biomarkers each year, new pharmacological and non-pharmacological methods of treatment and deceleration of disease progression remains unaddressed. Although the etiology of PD is not fully known, many mechanisms such as iron accumulation, disruption of calcium homeostasis, decreased level of trophic factors, mitochondrial dysfunction, dysfunction of ubiquitin-dependent protein degradation or neuroinflammation might be involved in PD pathogenesis (Castellani et al., 2000; Kalia and Lang, 2015). Moreover, neuroinflammation plays an especially important role in the dopaminergic neurons (DA-neurons) degeneration (Hirsch et al., 2012).

The main inflammatory factors–cytokines that play a role in immune system activation are divided into two groups: pro-inflammatory and anti-inflammatory factors. The most important and widely described pro-inflammatory cytokines are tumor necrosis factor α (TNF-α), interleukin 1β (IL-1β), and interferon γ (IFN-γ). Moreover, increased levels of interleukin 6 (IL-6), interleukin 10 (IL-10), interleukin 4 (IL-4), and interleukin 2 (IL-2) have been previously reported in the brain of PD patients (Imamura et al., 2003). During an acute bout of exercise, the production of IL-6 in skeletal muscles increases up to 100 times higher than during rest (measured before exercise) (Febbraio et al., 2003). This increase can be correlated to the elevated level of anti-inflammatory factors such as IL-1 receptor antagonist and IL-10 and decreased levels of TNF-α and IL-1β (Pedersen et al., 2003; Starkie et al., 2003; Suzuki, 2019; Suzuki et al., 2020).

Neuroinflammation is a characteristic of many neurological disorders, including neurodegenerative diseases such as PD or Alzheimer's disease. Activation of microglia in the *substantia nigra pars compacta* (SNpc) and striatum has been previously reported in animal models of PD, as well as in the brain of PD patients (McGeer et al., 1988; Benner et al., 2008). Chronic activation of microglia increases the level of TNF-α, IL-1β, IL-6, and IFN-γ, which are pro-inflammatory cytokines that play roles in DA-neuron degeneration (Ferrari et al., 2006; McCoy et al., 2006).

The main causes of microglial activation in PD are considered to be alpha-synuclein aggregates, except activation of microglia in PD astrogliosis is also observed (Wang et al., 2015). This activation is stimulated by the presence of pro-inflammatory factors such as lipopolysaccharides (LPS), IL-1β, and TNF-α (Saijo et al., 2009; Tanaka et al., 2013). Apart from the neuroinflammation in the central nervous system (CNS), many researchers have shown the activation of the peripheral immune system. Serum levels of cytokines such as IL-2, IL-10, IL-4, IL-6, IL-1β, TNF-α, and IFN-γ are elevated in PD patients (Brodacki et al., 2008; Reale et al., 2009). A meta-analysis including 2654 participants (1547 PD patients and 1107 healthy subjects) showed increased levels of IL-6, TNF-α, IL-1β, IL-10, and IL-2 in the peripheral blood of PD patients compared with the healthy subjects, supporting a possible role of peripheral immune system activation in the development of PD (Qin et al., 2016).

Due to its protective role, the blood-brain barrier (BBB) is composed of endothelial cells, pericytes, astrocytes, and the basement membrane, making it almost impossible for pathogens or peripheral immune cells to be transported from the peripheral system into the CNS (Ballabh et al., 2004; Hawkins and Davis, 2005). Similar to other neurodegenerative diseases, BBB breakdown has been reported in PD, causing increased infiltration of immune cells from the peripheral system into the CNS (Kortekaas et al., 2005). Under physiological conditions, cells such as T- and B-lymphocytes are hardly detectable in the CNS; however, in the brain of adeno-associated virus-mediated alpha-synuclein over-expressing mice, both cell types are elevated (Benner et al., 2008; Brochard et al., 2009). The main cause of the BBB breakdown is still unknown. Microglial overexpression and increased levels of pro-inflammatory factors in the brain might be responsible for the BBB disruption or activation of the peripheral immune system (Wang et al., 2015).

Brain-derived neurotrophic factor (BDNF)—another factor in neuroinflammation, has the ability to cross the BBB in both directions with the concentration gradient, and its concentration in the serum may reflect its level in the brain (Klein et al., 2011). BDNF plays a protective role against neurodegeneration via the activation of anti-apoptotic pathways (Klintsova et al., 2004; Numakawa et al., 2010). Mature BDNF (mBDNF) has a higher affinity to the tyrosine receptor kinase B (TrkB receptor), while pro-BDNF, the immature BDNF, more often interacts with p75 neurotrophin receptor (p75NTR) and activates pro-apoptotic pathways leading to cell apoptosis (Klintsova et al., 2004; Chen and Russo-Neustadt, 2005; Teng et al., 2005). Physical activity is known to increase BDNF levels. A meta-analysis of 11 articles with subjects suffering from neurological disorders presents that after aerobic training, the level of BDNF in the serum or plasma increases compared to the basal level (Mackay et al., 2017). The exact process leading to this increase caused by training is still unclear.

Endurance training is classified as a mode of exercises performed for a long duration with a low load work. It results in the improvement of the cardiovascular system (such as lower heart rate), respiratory system, lowering of glucose level, and increases in blood and plasma volume (Laursen and Jenkins, 2002; Hughes et al., 2017). Endurance training can be activities such as running, cycling, or swimming. Endurance exercises are characterized by repeats of isotonic contractions of large skeletal muscles and can be divided according to the intensity of performing exercises on low, moderate, and high-intensity exercises. Low-intensity exercises are described as work conduct below the anaerobic threshold <45% VO_2max_ (maximal oxygen uptake) or <70% of HR_max_ (maximal heart rate), moderate-intensity exercises are described at 50–60% of VO_2max_ or 50–75% of HR_max_ at the anaerobic threshold. High-intensity exercises are over an anaerobic level 80–90% of VO_2max_ or 85–95% of HR _max_ (Laursen and Jenkins, 2002). In this review, we focused on two types of endurance exercises: continuous exercise and training, characterized by continuous activity during the bout of exercise and interval exercise. The last modes of exercise are classified as a part of endurance exercises characterized by repeats of moderate or high-intensity bouts and low-intensity bouts or rest periods (Laursen and Jenkins, 2002). It is believed that interval endurance exercise can bring more benefits than continuous endurance exercise not only increased fat oxidation but also has a greater impact on the enhancement in endurance performance (Laursen and Jenkins, 2002).

Several meta-analyses published during the past few years focuses on the influence of many types of exercises, such as endurance exercises or resistance exercises, on the BDNF level in healthy young people and healthy older people, showing that performing physical activity leads to increases in the level of peripheral BDNF (Szuhany et al., 2015; Dinoff et al., 2017; Marinus et al., 2019). Moreover, it was previously discussed the effect of a single bout of exercise and regular performing exercise program on the BDNF level, showing a moderate effect size for a single bout of exercise on BDNF level, and a small effect size on the BDNF level after regular exercises (Szuhany et al., 2015). So far, only two meta-analyses show the influence of intensive training or rehabilitation on the BDNF level in the group of PD patients (Mackay et al., 2017; Hirsch et al., 2018). Most research focuses on the influence of endurance training on the BDNF level in healthy individuals. In the context of PD and the benefits of endurance training in the slowing down the disease progression or improvement of the life quality, not many promising studies were so far conducted. The last 2 years brought new light on introducing endurance training as a part of rehabilitation or the lifestyle in PD due to two promising randomized clinical trials (RCT) (Landers et al., 2019; O'Callaghan et al., 2019).

The aim of this narrative review is to focus on the relationship between a bout of endurance exercise, endurance training (continuous and interval), BDNF, and inflammatory status in the three subjects groups (young adults, older adults, and patients with PD) and to review the current state of knowledge about the possible causes of the differences in BDNF and inflammatory status response to a bout of endurance exercise and training. Furthermore, short, practical recommendations for PD patients were formulated for improving the efficacy of the training process during the rehabilitation. The following logic was used to include the three groups in this narrative review: (a) to present the different pattern of potential changes in inflammatory status and BDNF level in three populations different by age, health status, and training status; (b) attempt to explain the possible mechanism responsible for the following changes in the peripheral system in those groups.

## Method

The literature search was conducted in accordance with the Preferred Reporting Items for Systematic Reviews and Meta-Analyses (PRISMA) (Moher et al., 2009). An electronic search was performed using the databases PubMed/MEDLINE and Web of Science for all English-language articles in the fields of medicine, molecular biology, biochemistry, and health professions between January 2010 and January 2020. Keyword searches included combinations of the following terms: [(”healthy young adults” OR ”healthy older adults” OR “Parkinson”) AND (“endurance training” OR “interval training” OR “physical exercise”) AND (“inflammat^*^” OR “BDNF”) NOT (“Review”)]. Articles had to be original articles and not a meta-analysis or review. Subjects could not have other diseases such as diabetes, obesity, take any supplements, or smoke.

**Figure 1:**
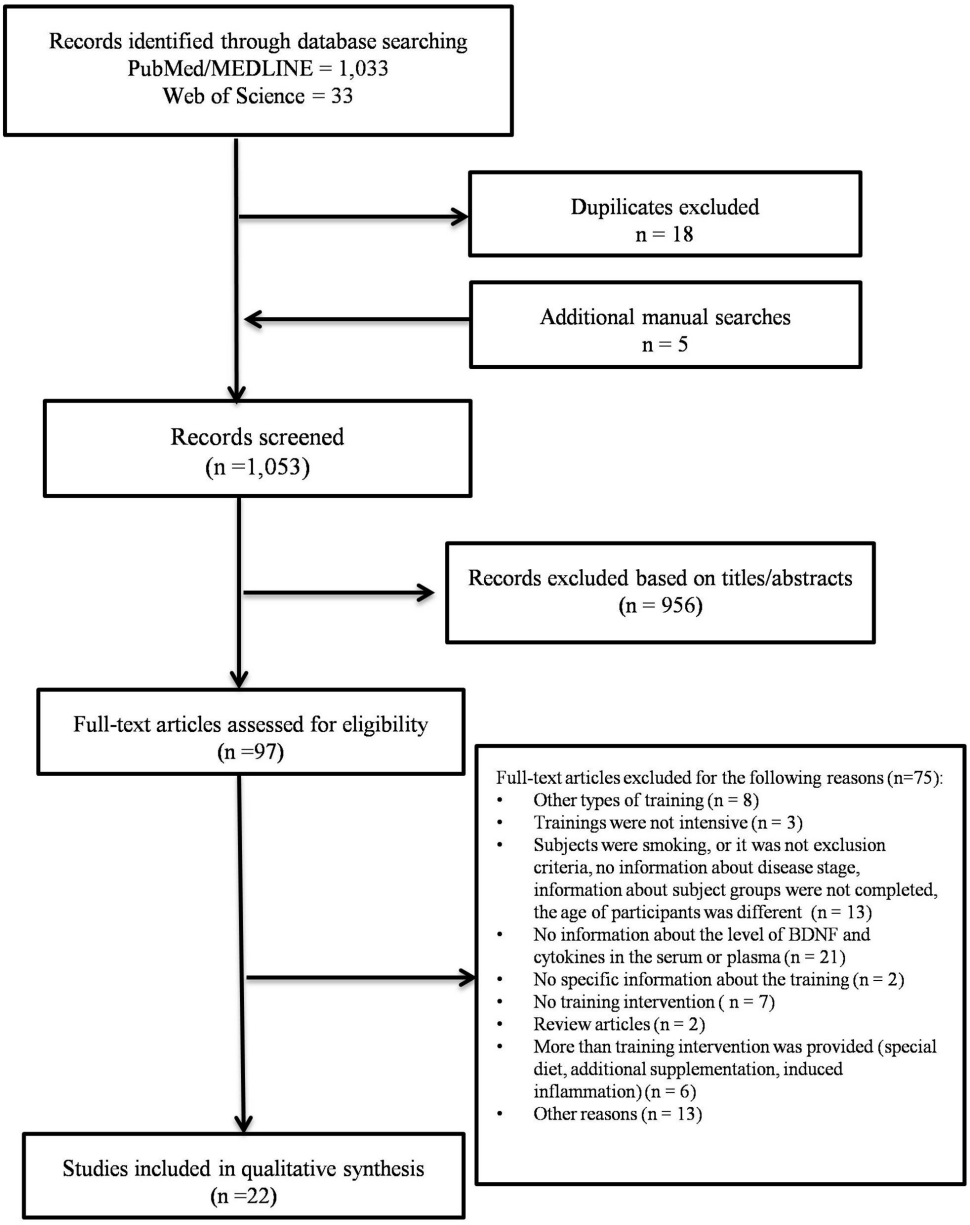
PRISMA flow diagram showing methodlogy for literature review and selection studies.

The following study criteria were met in this review: research was conducted in healthy young adults (18–40 years of age), healthy older adults (>50 years of age), or people with diagnosed idiopathic PD. Participants with PD had to be in the early or intermediate stage of the disease (Hoeh & Yahr (H&Y) scale 1-3). Articles with diagnosed metabolic, neurological, or psychiatric diseases were excluded. Publications without basic information such as participants' age and knowledge if they regularly performed a physical activity before the study or not, were rejected. Articles with resistance training or mixed variations of training were excluded. No information about the intervention or additional supplementation of vitamins, medication was excluded from this research. After reviewing the abstracts, titles, and full text, 22 articles were selected for the review (Figure 1). The obtained results were divided into two sections: endurance training and an acute bout of endurance exercise, to determine if the physical activity duration might play a significant role in the regulation of biochemical changes in the body.

Quality assessment: Studies qualified for this narrative review were applied for methodological quality and risk of bias according to the PEDro (Physiotherapy Evidence Database). This scale is accurate for the measurements of validation of clinical trials (Morton, 2009). Two authors independently screened and scored all 22 articles for assessment of the quality of each individual article.

## Results

### Included Studies and Quality Assessment

Twenty-two original studies with a total of 614 participants were included in this narrative review (Erickson et al., 2011; Ruscheweyh et al., 2011; Reihmane et al., 2013; Voss et al., 2013; Frazzitta et al., 2014; Leckie et al., 2014; Rinnov et al., 2014; Zoladz et al., 2014; de Gonzalo-Calvo et al., 2015; Marquez et al., 2015; Wagner et al., 2015; Cabral-Santos et al., 2016; Hwang et al., 2016; Kaspar et al., 2016; Maass et al., 2016; Marusiak et al., 2016; Schega et al., 2016; Brown et al., 2018; Slusher et al., 2018; Abkenar et al., 2019; Landers et al., 2019; O'Callaghan et al., 2019). The search strategy presented 1,033 records from PubMed/MEDLINE, 33 records from Web of Science, and five articles were identified through other sources. According to the previous research, a performed search in two chosen databases could allow us to find about 85% of the literature on this topic (Bramer et al., 2017). After removing duplicates, 1,053 records were screened. Nine hundred and fifty-sixth articles were excluded based on titles and abstracts and the full texts of 97 articles were assessed for eligibility. After reviewing the full-text articles, 22 studies were chosen for further analysis in this review while 75 articles were excluded (Figure 1).

The average quality assessment of the 22 included studies determined by using PEDro criteria was 4.36 out of 10. The results of the quality assessments were presented in Table 1. Five studies included in this research were defined as RCT (Erickson et al., 2011; Wagner et al., 2015; Schega et al., 2016; Landers et al., 2019; O'Callaghan et al., 2019), one study was defined as control trial (CT) (Maass et al., 2016), ten studies qualified as quasi-experimental studies (QE) (Ruscheweyh et al., 2011; Voss et al., 2013; Frazzitta et al., 2014; Leckie et al., 2014; Rinnov et al., 2014; Marquez et al., 2015; Kaspar et al., 2016; Marusiak et al., 2016; Slusher et al., 2018; Abkenar et al., 2019) and six as pre-post studies (Reihmane et al., 2013; Zoladz et al., 2014; de Gonzalo-Calvo et al., 2015; Cabral-Santos et al., 2016; Hwang et al., 2016; Brown et al., 2018). The highest quality score (7-10 points according to the criteria) had five studies (Erickson et al., 2011; Frazzitta et al., 2014; Schega et al., 2016; Landers et al., 2019; O'Callaghan et al., 2019). Eleven studies performed the random allocation for subjects (criteria 2) (Erickson et al., 2011; Ruscheweyh et al., 2011; Voss et al., 2013; Frazzitta et al., 2014; Hwang et al., 2016; Kaspar et al., 2016; Maass et al., 2016; Schega et al., 2016; Abkenar et al., 2019; Landers et al., 2019; O'Callaghan et al., 2019), five studies performed blinding for all subjects (criteria 5) (Voss et al., 2013; Frazzitta et al., 2014; Schega et al., 2016; Landers et al., 2019; O'Callaghan et al., 2019), and only three studies performed blinding of assessors for researchers (criteria 7) (Frazzitta et al., 2014; Landers et al., 2019; O'Callaghan et al., 2019). Three criteria (criteria 8, criteria 10, and criteria 11) were fulfilled in 90–100% of trials (Erickson et al., 2011; Ruscheweyh et al., 2011; Reihmane et al., 2013; Voss et al., 2013; Frazzitta et al., 2014; Leckie et al., 2014; Rinnov et al., 2014; Zoladz et al., 2014; de Gonzalo-Calvo et al., 2015; Marquez et al., 2015; Wagner et al., 2015; Cabral-Santos et al., 2016; Hwang et al., 2016; Kaspar et al., 2016; Maass et al., 2016; Marusiak et al., 2016; Schega et al., 2016; Brown et al., 2018; Slusher et al., 2018; Abkenar et al., 2019; Landers et al., 2019; O'Callaghan et al., 2019). Only one study performed blinding of therapists to aims and hypotheses (criteria 6) (Landers et al., 2019).

**Table 1:** Quality assessment of the included studies using PEDro checklist.

**Study**	**Design, quality**	**C1**	**C2**	**C3**	**C4**	**C5**	**C6**	**C7**	**C8**	**C9**	**C10**	**C11**	**Total**
Brown et al. (2018)	Pre-post	N	N	N	N	N	N	N	Y	N	Y	Y	3
Cabral-Santos et al. (2016)	Pre-post	N	N	N	N	N	N	N	N	N	Y	Y	2
de Gonzalo-Calvo et al. (2015)	Pre-post	Y	N	N	N	N	N	N	Y	N	Y	Y	4
Reihmane et al. (2013)	Pre-post	N	N	N	N	N	N	N	Y	N	Y	Y	3
Kaspar et al. (2016)	QE	N	Y	N	N	N	N	N	Y	N	Y	Y	4
Abkenar et al. (2019)	QE	Y	Y	N	N	N	N	N	Y	N	Y	Y	5
Wagner et al. (2015)	RCT	N	N	N	N	N	N	N	Y	N	Y	Y	3
Rinnov et al. (2014)	QE	Y	N	N	Y	N	N	N	Y	N	Y	Y	4
Zoladz et al. (2014)	Pre-post	Y	N	N	N	N	N	N	Y	N	Y	Y	4
Landers et al. (2019)	RCT	Y	Y	Y	Y	Y	Y	Y	Y	N	Y	Y	10
Hwang et al. (2016)	Pre-post	N	Y	N	Y	N	N	N	Y	N	Y	Y	5
Marquez et al. (2015)	QE	N	N	N	N	N	N	N	Y	N	Y	Y	3
Slusher et al. (2018)	QE	Y	N	N	N	N	N	N	Y	N	Y	Y	4
O'Callaghan et al. (2019)	RCT	Y	Y	Y	N	Y	N	Y	N	N	Y	Y	8
Leckie, et al. (2014)	QE	Y	N	N	Y	N	N	N	N	N	Y	Y	4
Erickson et al. (2011)	RCT	Y	Y	Y	Y	N	N	N	Y	N	Y	Y	7
Frazzitta et al. (2014)	QE	Y	Y	Y	Y	Y	N	Y	Y	N	Y	Y	9
Ruscheweyh et al. (2011)	QE	N	Y	N	Y	N	N	N	Y	N	Y	Y	5
Voss et al. (2013)	QE	Y	Y	N	Y	Y	N	N	Y	N	Y	Y	6
Maass et al. (2016)	CT	Y	Y	N	Y	N	N	N	Y	N	Y	Y	6
Schega et al. (2016)	RCT	Y	Y	N	Y	Y	N	N	Y	N	Y	Y	7
Marusiak et al. (2016)	QE	Y	N	N	N	N	N	N	Y	N	Y	Y	4

*C1: eligibility criteria were specified; C2: subjects were randomly allocated to groups; C3: allocation was concealed; C4: groups were similar at baseline; C5: all subjects were blinded; C6: all therapist were blinded; C7: all assessors were blinded; C8: at least one key outcome measured was obtained from more than 85% of the subjects; C9: intention to treat; C10: statistical comparison was reported for at least once; C11: points measures of variability was reported for at least one outcome. Criteria 2-11 scored; Y–yes, criteria is satisfied; N–no, criteria was not satisfied*.

### Inflammatory Status After a Single Bout of Endurance Exercise and Endurance Training in Healthy Young Subjects

Despite the training time, age, and training status of subjects, chronic endurance training may involve a similar inflammatory response, allowing the detection of certain trends associated with the response of the inflammatory system to a single bout of endurance exercise. In this review, eight articles with healthy young adults were analyzed in the context of the influence of an acute bout of endurance exercise and endurance training on inflammatory cytokines (Reihmane et al., 2013; Rinnov et al., 2014; de Gonzalo-Calvo et al., 2015; Wagner et al., 2015; Cabral-Santos et al., 2016; Kaspar et al., 2016; Brown et al., 2018; Abkenar et al., 2019). Seven studies with a total of 133 young adults (age 18–40) showed the influence of acute bout of endurance exercise on cytokines such as TNF-α, IL-6, IL-10, interleukin 15 (IL-15), IL-1β, morphological parameters (leukocytes, neutrophils, lymphocytes, monocytes), and CRP (C-reactive protein) (Table 2; Reihmane et al., 2013; Rinnov et al., 2014; de Gonzalo-Calvo et al., 2015; Cabral-Santos et al., 2016; Kaspar et al., 2016; Brown et al., 2018; Abkenar et al., 2019). Four studies present only measurements before and after the intervention (pre-post studies) (Reihmane et al., 2013; de Gonzalo-Calvo et al., 2015; Cabral-Santos et al., 2016; Brown et al., 2018) and three studies are described as QE (Rinnov et al., 2014; Kaspar et al., 2016; Abkenar et al., 2019). Only two studies included the results from a single bout of endurance exercise following a program of endurance training and after the endurance training program completion (Rinnov et al., 2014; Abkenar et al., 2019).

**Table 2:** Changes in the inflammatory status after the acute bout of endurance exercise.

**Mode of exercise**	**Subjects**	**Sampling points**	**Material**	**Inflammatory parameters**			**References**
				**Immediately after the exercise**	**At rest level (60 min−48 h after the exercise)**		
Single session of high intensity endurance walking at 90% of HR _max_	20 healthy men 24.1 ± 1.8 yr	Before and after the exercise	PBMCs	IL-1β IL-18 ns	-		Abkenar et al., 2019
Single session of moderate intensity walking at 70% of HR _max_	20 healthy men 23.65 ± 2.43 yr	Before and after the exercise	PBMCs	IL-1β IL-18 ns	-		Abkenar et al., 2019
One session of high intensity interval walking exercise at 80% of VO_2max_	17 active men 22.6 ± 4.6 yr	Before, immediately after, 48 h after the exercise	Plasma	IL-6↑ TNF-α↑	IL-6 ↓		Brown et al., 2018
One session of continuous moderate walking exercise at 60% of VO_2max_	17 active men 22.6 ± 4.6 yr	Before, immediately after, 48 h after the exercise	Plasma	IL-6↑ TNF-α↑	IL-6 ↓		Brown et al., 2018
One session of high intensity interval training cycling	9 healthy men 20.9 ± 0.9 yr	Before, immediately after and 2 day after the exercise	Plasma	IL-1β, IL-6, IL-10, CRP ns	IL-1β, IL-6, IL-10, CRP ns		Kaspar et al., 2016
One session of moderate endurance cycling at 62.5% of HR_max_	9 healthy men 20.9 ± 0.9 yr	Before, immediately after and 2 day after the exercise	Plasma	IL-1β, IL-6, IL-10, CRP ns	IL-1β, IL-6, IL-10, CRP ns		Kaspar et al., 2016
One session of high intensity intermittent exercises–run distance 1.25 km	10 active men 25.22 ± 1.74 yr	Rest, immediately after, 60 min after the exercise	Plasma/serum	IL-6↑ IL-10↑	IL-6↓ IL-10 ↑		Cabral-Santos et al., 2016
One session of high intensity intermittent exercises–run distance 2,5 km	10 active men 25.22 ± 1.74 yr	Rest, immediately after, 60 min after the exercise	Plasma/serum	IL-6↑ IL-10↑	IL-6↓ IL-10 ↓		Cabral-Santos et al., 2016
10 km run	9 amateur runners men 39.1 ± 2.2 yr	Before, immediately after, 24 h after, 72 h after the exercise	Whole blood	leukocyte, neutrophils, monocytes, lymphocytes ↑	24 h after: leukocyte, neutrophils, monocytes, lymphocytes↓		de Gonzalo-Calvo et al., 2015
Half-marathon	9 amateur runners men 39.1 ± 2.2 yr	Before, immediately after, 24 h after, 72 h after the exercise	Whole blood	leukocyte, neutrophils, lymphocytes, IL-6, IL-10 ↑, monocytes ↓	24 h after: leukocyte, neutrophils, monocytes, lymphocytes, IL-6, IL-10 ↓ CRP ↑	72 h after: monocytes ↑ CRP↓	de Gonzalo-Calvo et al., 2015
Marathon	9 amateur runners men 39.1 ± 2.2 yr	Before, immediately after, 24 h after, 72 h after the exercise	Whole blood	Immediately after: leukocyte, neutrophils, monocytes, IL-6, IL-10, IL-8 ↑ lymphocytes ↓	24 h after: leukocyte, neutrophils, monocytes, IL-6, IL-10, IL-8 ↓ lymphocytes, CRP ↑	72 h after: monocytes↓, IL-8 ↓ CRP↓	de Gonzalo-Calvo et al., 2015
Marathon	18 marathon runners 26.0 ± 5.0 yr	Before, after the race, 28 h after the exercise	Serum	IL-6 ↑, TNF-α ↑	28 h after the race” IL-6 ns, TNF-α ns		Reihmane et al., 2013
Half-marathon	22 half marathon runners 27.0 ± 5.0 yr	Before, after the race, 28 h after the exercise	Serum	IL-6 ns, TNF-α ns	28 h after the race” IL-6 ns, TNF-α ns		Reihmane et al., 2013
3 h of acute endurance exercise–cycling at 60% of VO_2max_	8 healthy men 27.0 ± 3.7 yr	Before and after the exercise	Muscle biopsy, plasma, mRNA	IL-15 ns	-		Rinnov et al., 2014

*HR, heart rate; VO_2max_, maximal oxygen uptake; yr, years; ns, not statistical; IL, interleukin; TNF, tumor necrosis factor; PBMC, peripheral blood mononuclear cell; mRNA, messenger RNA. ↑, statistical increased level; ↓, statistical decreased level*.

Most research has shown elevated levels of IL-6, IL-10, and TNF-α after interval endurance training and after continuous endurance training (Reihmane et al., 2013; de Gonzalo-Calvo et al., 2015; Cabral-Santos et al., 2016; Brown et al., 2018). No significant changes in the inflammatory response were observed after a single bout of high-intensity interval and continuous endurance exercise (90% of HR_max_) (20 healthy men with an average age of 24.1 ± 1.8 years and nine men with an average age of 20.9 ± 0.9 years) in two studies and after acute moderate-intensity endurance exercise (at 62.5% of HR_max_ and 70% of HR_max_) in two studies (20 healthy men with an average age of 23.65 ± 2.43 years and nine men with average age 20.9 ± 0.9 years) (Kaspar et al., 2016; Abkenar et al., 2019). Measurements 48 h after a bout of high-intensity interval exercise and moderate-intensity continuous exercise completion showed decreased levels of IL-6 in the plasma of 17 men with an average age of 22.6 ± 4.6 years in both interventions (Brown et al., 2018).

Sixty minutes after acute endurance running distance (2.5 km) (10 men with an average age of 25.22 ± 1.74 years) and a short distance running (1.25 km) in the same subjects group, IL-6 was decreased compared to the level immediately after the exercise, while IL-10 was still elevated after the short distance running (1.25 km) and decreased after a longer distance (2.5 km) (Cabral-Santos et al., 2016). No significant changes were observed 1 day after a marathon and half-marathon in one study with 18 marathon runners (average age 26.0 ± 5.0 years) and 22 half-marathon runners (average age 27.0 ± 5.0 years) (Reihmane et al., 2013). Moreover, after the half-marathon, no changes in the levels of IL-6 and TNF-α were observed immediately after the race (Reihmane et al., 2013). No changes in the serum and muscle level of IL-15 were observed in the group eight subjects (age 27.0 ± 3.7 years) after 3 h of cycling run at 60% of VO_2max_ (Rinnov et al., 2014). One article showed changes in the parameters of white blood cells such as leukocytes, neutrophils, and monocytes after endurance exercise such as for a marathon on nine amateur runners (average age 39.1 ± 2.2 years) (de Gonzalo-Calvo et al., 2015). Immediately after a marathon, inflammatory parameters such as leukocytes, neutrophils, monocytes, IL-6, interleukin 8 (IL-8), and IL-10 increased, while the lymphocyte count decreased. Twenty-four hours after the run, all those parameters decreased, recurring to the basal line, while lymphocytes and CRP increased. At 72 h after the run CRP, monocytes and, IL-8 levels returned to basal values (de Gonzalo-Calvo et al., 2015). The same research team showed that immediately after a half marathon (9 subjects with an average age of 39.1 ± 2.2 years), leukocytes, neutrophils, IL-6, IL-10, and lymphocytes increased while monocytes decreased. After 24 h, all the parameters decreased except CRP, which increased. After 72 h, the run completion monocytes were elevated, while CRP decreased. A 10 km run performed by nine amateur runners (average age 39.1 ± 2.2 years) resulted in elevated levels of leukocytes, neutrophils, monocytes, and lymphocytes immediately after the run. A decreased level of those proteins was reported within the subsequent 24 h (de Gonzalo-Calvo et al., 2015).

Three studies with a total of 57 young participants were analyzed in the context of the influence of endurance training on inflammation (Table 3). Two studies are described as QE studies (Rinnov et al., 2014; Abkenar et al., 2019) and one study is designed as RCT (Wagner et al., 2015). Moderate-intensity endurance training (50–70% of HR_max_) results in decreased levels of IL-1β and interleukin 18 (IL-18) in 20 healthy subjects (age 23.65 ± 2.43 years), while high-intensity interval endurance training (70–90% of HR_max_ and 80% of VO_2max_) leads to the increased level of IL-1β, IL-18, TNF-α in peripheral blood mononuclear cells (PBMCs) and serum (Wagner et al., 2015; Abkenar et al., 2019). Increased level of IL-15 in muscle with no changes in the serum were observed after 12 weeks of endurance training in 10 men (age 30.5 ± 5.5 years) (Rinnov et al., 2014).

**Table 3:** Changes in the inflammatory status after the endurance training.

**Mode of training**	**Subjects**	**Sampling points**	**Material**	**Inflammatory parameters**		**References**
				**Immediately after the training**	**At rest level after the training**	
12 weeks of high intensity interval training at 70–90% of HR_max_	20 healthy men 24.1 ± 1.8 yr	Before and after the training	PBMCs	IL-1β ↑ IL-18 ↑	-	Abkenar et al., 2019
12 weeks of moderate intensity training at 50–70% of HR_max_	20 healthy men 23.65 ± 2.43 yr	Before and after the training	PBMCs	IL-1β ↓ IL-18 ↓	-	Abkenar et al., 2019
6-week intense aerobic exercise at 80% of VO_2max_	17 healthy men 25.0 ± 3.3 yr	Before and after the training	Serum	TNF-α ↑, IL-6 ns	-	Wagner et al., 2015
12 weeks of endurance training	10 trained men 30.5 ± 5.5 yr	Before and after the training	Muscle biopsy, plasma, mRNA	Muscle: IL-15 ↑	-	Rinnov et al., 2014
8 weeks of moderate-intensity training–cycling at 60–75% of HR_max_	12 PD patients 70 ± 3 yr H&Y scale 2.3 ± 0.2	Before and after the training	Serum	TNF-α ↓	-	Zoladz et al., 2014
8 weeks of aerobic training–treadmill at 70–80% of HR_max_	14 PD patients 63.5 ± 10.9 yr H&Y scale 2.0 ± 0.53	Before, immediately after and 6 months after the training	Serum	TNF-α ns IL-10 ↑	TNF-α ↑	Landers et al., 2019
8 weeks of low intensity training	13 PD patients 64.6 ± 6.0 yr H&Y scale 2.0 ± 0.68	Before, immediately after and 6 months after the training	Serum	IL-10 ↑	IL-6 ↓	Landers et al., 2019

*HR, heart rate; VO_2max_, maximal oxygen uptake; yr, years; ns, not statistical; IL, interleukin; TNF, tumor necrosis factor; PBMC, peripheral blood mononuclear cell; mRNA- messenger RNA; PD, Parkinson's disease'; H&Y scale, Hoehn & Yahr scale. ↑, statistical increased level; ↓, statistical decreased level*.

### Inflammatory Status After Endurance Training in Parkinson's Disease Patients

Two studies have described the influence of prolonged endurance intervention on the inflammatory status in a total of 39 PD patients (Table 3). One study was described as a pre-post study and one as RCT (Zoladz et al., 2014; Landers et al., 2019). Zoladz et al. showed that 8 weeks of moderate-intensity interval training (cycling at 60-75% of HR_max_) decreases TNF-α levels in the serum of 12 PD patients (age 70.0 ± 3 years, H&Y scale 2.3 ± 0.2); however, Landers et al. showed no difference in the plasma level of that cytokine before and after 8 weeks of high-intensity aerobic training (treadmill walking at 70-80% of HR_max_) in a group of 14 PD patients (age 63.5 ± 10.9 years, H&Y scale 2.0 ± 0.53) (Zoladz et al., 2014; Landers et al., 2019). The only observation was that TNF-α increased 6 months after the end of the training. A decreased level of IL-6 was reported 6 months after the high-intensity training had ended in the group of 14 PD patients. The only significantly changed cytokine immediately after the training was IL-10 in the high-intensity and low-intensity training groups. Additionally, the IL-10/TNF-α ratio increased immediately after the completion of both training events (Landers et al., 2019).

### BDNF Level After an Acute Bout of Endurance Exercise and Endurance Training in Healthy Subjects

Four studies with a total of 81 young participants were included in the analysis of the influence of acute bout of endurance exercise on BDNF level (Table 4). Two studies were described as a pre-post study (Cabral-Santos et al., 2016; Hwang et al., 2016) and two as QE studies (Marquez et al., 2015; Slusher et al., 2018). The time and mode of training and subjects are particularly important factors during BDNF secretion. A single bout of high-intensity endurance exercise (70–90% of HR_max_ and 70–100% of VO_2max_) causes only a temporary increase in the BDNF level in the serum and plasma of 81 young, healthy people (age range 18–30 years) (Marquez et al., 2015; Cabral-Santos et al., 2016; Hwang et al., 2016; Slusher et al., 2018). Measurements performed 20-30 min after exercise completion showed a significantly decreased BDNF level, compared with the baseline level (Marquez et al., 2015; Cabral-Santos et al., 2016; Hwang et al., 2016).

**Table 4:** Changes in the BDNF level after the acute bout of endurance exercise.

**Mode of exercise**	**Subject**	**Sampling points**	**Material**	**BDNF level**		**References**
				**Immediately after the exercise**	**At rest level after the exercise**	
Single session of endurance interval exercise at 85–90% of VO_2max_	29 healthy adults 18-29 yr	Before, immediately after, 30 min after the exercise	Serum	BDNF ↑	BDNF ↓	Hwang et al., 2016
Single session of endurance interval exercise at 70% at 90% of maximal work load	8 active men 28 ± 5 yr	Before, immediately after, 20 min after the exercise	Serum	BDNF ↑:	BDNF ↓	Marquez et al., 2015
Single session of continuous cycling exercise at 70% of max work rate	21 active men 27 ± 4 yr	Before, immediately after, 20 min after the exercise	Serum	BDNF ↑	BDNF ↓	Marquez et al., 2015
One session of high intensity interval exercises–run distance 1.25 km	10 active men 25.22 ± 1.74 yr	Rest, immediately after, 60 min after the exercise	Plasma/serum	BDNF ↑	BDNF ↓	Cabral-Santos et al., 2016
One session of high intensity intermittent exercises–run distance 2.5 km	10 active men 25.22 ± 1.74 yr	Rest, immediately after, 60 min after the exercise	Plasma/serum	BDNF ↑	BDNF ↓	Cabral-Santos et al., 2016
Super maximal single session of endurance interval exercise at 170% of VO_2peak_	13 healthy men 23.62 ± 1.06 yr	Before and after the exercise	Plasma	BDNF ↑	-	Slusher et al., 2018
Single session of high intensity exercise at 85% HR _max_	17 PD patients Immediate start group: 68.8 ± 7.9 yr H&Y scale 2.33 ± 0.47 Delayed start group: 69.0 ± 6.63 yr H&Y scale 2.43 ± 0.49	Before and after the exercise	Serum	BDNF ns	-	O'Callaghan et al., 2019
Single session of moderate intensity exercise at 60–80% HR _max_	13 PD patients 70.4 ± 7.2 yr H&Y scale 2,07 ± 0.27	Before and after the exercise	Serum	BDNF ns	-	O'Callaghan et al., 2019

*HR, heart rate; VO_2max_, maximal oxygen uptake; yr, years; ns, not statistical; BDNF, brain derived neurotrophic factor; PD, Parkinson's disease; H&Y scale, Hoehn & Yahr scale. ↑, statistical increased level; ↓, statistical decreased level*.

Seven studies with a total of 296 older people and 17 younger men were analyzed in the context of endurance training in the regulation of BDNF level (Table 5). Three studies were designed as RCT (Erickson et al., 2011; Wagner et al., 2015; Schega et al., 2016), one as a clinical trial (Maass et al., 2016) and three as QE studies (Ruscheweyh et al., 2011; Voss et al., 2013; Leckie et al., 2014). After long moderate-intensity endurance training (50-75% of HR_max_) lasting 1 year, elevated levels of BDNF were reported in the group of 47 sedentary older adults (age 67.23 ± 5.39 years) and the group of 21 older adults (62.5 ± 6.4 years) after 6 months of gymnastic intervention (at 30-40% HR_max_) but not in the group of 20 older people (60.1 ± 6.2 years) after 6 months of moderate-intensity Nordic walking intervention (at 50-60% of HR_max_) (Ruscheweyh et al., 2011; Leckie et al., 2014). Similar observations were reported after 7 weeks of low-intensity (stretching exercises) and moderate-intensity endurance training (walking at 50-60% of HR_max_) in the two groups of 60 older adults (age 66.6 ± 5.8 years and 65.5 ± 5.44 years) when BDNF level was reported higher after both interventions compared to the basal level (Erickson et al., 2011). No changes in BDNF level were observed after 4 weeks of moderate-intensity endurance training (cycling at 65–75% of HR_max_) in 18 older people (age 66.4 ± 3.3 years), and after 12 weeks of intensive endurance training (interval running on the treadmill at ≥ 65% of HR_max_) in the group of 40 sedentary older people (age 68.4 ± 4.3 years) (Maass et al., 2016; Schega et al., 2016). Moreover, a 1-year intervention of moderate-intensity endurance training (walking at 60-75% of HR_max_) in the group of 30 older adults (age 67.3 ± 5.8 years) did not show the significant changes in the serum level of BDNF (Voss et al., 2013). One study with 17 men (age 25.0 ± 3.3 years) showed that 6 weeks of high-intensity endurance training (HR_max_ ≥ 65) decreases the BDNF level in the serum (Wagner et al., 2015).

**Table 5:** Changes in the BDNF level after the chronic endurance training.

**Mode of training**	**Subjects**	**Sampling points**	**Material**	**BDNF level**		**References**
				**Immediately after the training**	**At rest level after the training**	
12 weeks intensive endurance training on treadmill (aerobic and stretching exercises) at ≥ 65% of HRmax	40 sedentary healthy older adults 68.4 ± 4.3 yr	Before and after the training	Serum and plasma	BDNF ns	-	Maass et al., 2016
4 weeks of aerobic training on bicycle ergometer at 65–75% of HR_max_	18 older adults 66.4 ± 3.3 yr	Before and after the training	Serum	BDNF ns	-	Schega et al., 2016
6-week intense aerobic exercise at about 77% of VO_2max_	17 healthy men 25.0 ± 3.3 yr	Before and after the training	Serum	BDNF ↓	-	Wagner et al., 2015
1-year of moderate intensity walking training at 60–75% of HR_max_	47 older adults 67.23 ± 5.39 yr	Before and after the training	Serum	BDNF ↑	-	Leckie et al., 2014
1 year of an aerobic walking training at 60–75% of HR_max_	30 older adults 67.3 ± 5.8 yr	Before and after the training	Serum	BDNF ns	-	Voss et al., 2013
7 weeks of walking at 50–60% of HR_max_	Aerobic training: 60 older adults 66.6 ± 5.8 yr	Before and after the training	Serum	BDNF ↑	-	Erickson et al., 2011
7 weeks of stretching	stretching training: 60 older adults 65.5 ± 5.44 yr	Before and after the training	Serum	BDNF ↑	-	Erickson et al., 2011
Nordic walking at 50–60% of HR_max_	Nordic walking: 20 older adults 60.1 ± 6.2yr	Before and after the training	Serum	BDNF ns	-	Ruscheweyh et al., 2011
Gymnastic at 30–40% of HR_max_	21 older adults 62.5 ± 6.4yr	Before and after the training	Serum	BDNF ↑	-	Ruscheweyh et al., 2011
8 weeks of moderate intensity training—cycling at 60–75% of HR_max_	12 PD patients 70 ± 3 yr H&Y scale 2.3 ± 0.2	Before and after the training cycle	Serum	BDNF ↑	-	Zoladz et al., 2014
8 weeks of moderate-intensity endurance training—cycling at 60–70% of HR_max_	11 PD patients 70.54 ± 9.56 yr H&Y scale 2.32 ± 0.68	1–3 days before training and 6–10 days after the training	Serum	BDNF ↑	-	Marusiak et al., 2016
8 weeks of aerobic training—treadmill at 70–80 of HR_max_	14 PD patients 63.5 ± 10.9 yr H&Y scale 2.0 ± 0.53	Before, 48–72 h after and 6 months after the training	Plasma	BDNF ↑	BDNF ↓	Landers et al., 2019
8 weeks of low intensity training	13 PD patients 64.6 ± 6.0 yr H&Y scale 2.0 ± 0.68	Before, immediately after and 6 months after the training	Plasma	BDNF ↑	BDNF ↓	Landers et al., 2019
12 weeks of high intensity training at 85% HR_max_	17 PD patients Immediate start group: 68.8 ± 7.9 yr H&Y scale 2.33 ± 0.47 Delayed start group: 69.0 ± 6.63 yr H&Y scale 2.43 ± 0.49	Before and after the training	Serum	BDNF ↑	-	O'Callaghan et al., 2019
12 weeks of moderate intensity exercise at 60–80% HR_max_	13 PD patients 70.4 ± 7.2 yr H&Y scale 2.07 ± 0.27	Before and after the training	Serum	BDNF ns	-	O'Callaghan et al., 2019
4 weeks of moderate intensity endurance training/rehabilitation at 60% of HR_max_	14 PD patients 67 ± 5 yr H&Y scale 1–1.5	Before and after the training	Serum	BDNF ↑	-	Frazzitta et al., 2014

*HR, heart rate; VO_2max_, maximal oxygen uptake; yr, years; ns, not statistical; BDNF, brain derived neurotrophic factor; PD, Parkinson's disease; H&Y scale, Hoehn & Yahr scale. ↑, statistical increased level; ↓, statistical decreased level*.

### BDNF Level After a Single Bout of Endurance Exercise and Endurance Training in the Parkinson's Disease

Five studies with a total of 95 participants with diagnosed PD were analyzed in this review (Table 5). All patients were in the early or intermediate stage of the disease (H&Y scale 1-3). Two studies were designed as RCT (Landers et al., 2019; O'Callaghan et al., 2019), and one as a randomized study (Frazzitta et al., 2014). One study was described as QE (Marusiak et al., 2016) study and one as a pre-post study (Zoladz et al., 2014). Thus far, only one study has shown the influence of a single session of interval endurance training and moderate-intensity endurance exercises on the BDNF level in 17 PD patients with intermittent disease stage (O'Callaghan et al., 2019). O'Callaghan et al. showed no significant changes in the serum BDNF level after both training types (O'Callaghan et al., 2019). In the PD groups, an increased level of BDNF after 8 weeks of moderate-intensity interval endurance training (60-75% of HR_max_) was described in two studies (12 people with PD, average age 70 years, H&Y scale 2.3 ± 0.2 and 11 people with PD, average age 70.54 years, H&Y scale 2.32 ± 0.68) (Zoladz et al., 2014; Marusiak et al., 2016). Elevated levels of BDNF in the plasma after 8 weeks of high-intensity endurance training (70–80% of HR_max_, 14 people with PD, average age 63.5 years, H&Y scale 2.0 ± 0.53) and 8 weeks of low-intensity endurance training (13 people with PD, average age 64.6 years, H&Y scale 2.0 ± 0.68) was presented by Landers et al.; however, the level of BDNF drastically decreased below baseline 6 months after the end of the training cycle in both groups (Landers et al., 2019). One study with 12 weeks of high-intensity interval endurance training (cycling at 85% of HR_max_) and moderate-intensity endurance training (60–80% of HR_max_) showed that high-intensity interval endurance training improved the BDNF level in the serum of 17 people with PD (average age 68.9 years and H&Y scale 2.38), while moderate-intensity training showed no difference after 12 weeks of training in the serum of 13 people with PD (average age 70.4 years and H&Y scale 2.07) (O'Callaghan et al., 2019). A randomized study performed at the group of 15 participants in the early stage of PD (H&Y 1–1.5; age 65 ± 5 years), who performed intense rehabilitation consist of moderate-intensity endurance training for 4 weeks (at 60% of HR_max_) showed an increased level of BDNF in the serum comparing to the basal level and control group (without intervention) (Frazzitta et al., 2014).

## Discussion

The main aim to conduct this narrative review was to summarize the current state of knowledge about the influence of an endurance bout of exercise and endurance training on the inflammatory status and BDNF level in young, healthy adults, older healthy adults, and in people with PD. The analysis shows the differences between responses on the intervention such as endurance training in three groups who are different by age and the health status. It is well-known that in the group of young, healthy people, mechanisms responsible for the response to intensive training might be different from the group of older healthy people and people with PD. As it was previously mentioned, in many publications, one of the main factors which influence the response to the endurance training might have been the brain condition such as the stage of neurons damaged caused by aging processes (Erickson et al., 2011). The effect of endurance training might be observed differently in the population of young people and different in the population of older people (Erickson et al., 2011; Ruscheweyh et al., 2011; Voss et al., 2013; Leckie et al., 2014; Wagner et al., 2015; Maass et al., 2016; Schega et al., 2016). It is important to mention that completely different results after the training intervention can be observed in the group of people with PD than in older adults, not only young adults, due to possible biochemical changes caused by disease progression or the possible involvement of other pathways in response to the exercises. In this narrative review, we decided to analyze the newest scientific reports from the past 10 years to summarize the recent findings. During the past 10 years the state of knowledge about the training intervention on biochemical changes in the PD is rapidly developing and every year more researchers present their findings of the improvement of endurance training on brain activity, neuroplasticity, and cardiorespiratory system (Kelly et al., 2017; Harvey et al., 2018; Malczynska et al., 2019). Even though before 2010 many publications included studies about the influence of endurance training and bout of endurance exercise on neurotrophic factors in the healthy subjects, we decided to not repeat the analysis of those finding because the results were very well-described and presented in systematic reviews and meta-analysis, with healthy young people and healthy older people, published in previous years (Szuhany et al., 2015; Dinoff et al., 2017; Marinus et al., 2019).

### Inflammatory Status After Single Bout of Endurance Exercise and Endurance Training in Healthy Young Subjects

In this narrative review, we analyzed 10 articles (eight with healthy subjects and two with PD participants) from the past 10 years with a total of 190 healthy participants and 39 people with PD, focused on the inflammatory response on an acute bout of endurance exercise and endurance training. Most studies have shown increases in TNF-α not only after an acute bout of endurance exercise but also after endurance training (Reihmane et al., 2013; Wagner et al., 2015; Brown et al., 2018). Moreover, it has been shown that immediately after the completion of acute endurance exercise, cytokines such as IL-6, IL-10, and TNF-α increase temporally, then coming back to their basal level within 60 min (de Gonzalo-Calvo et al., 2015; Cabral-Santos et al., 2016; Brown et al., 2018). One study compared a single bout of endurance exercise with two different intensities: moderate-intensity (50-70% of HR_max_) and high-intensity (70-90 % of HR_max_) and 12 weeks of the same intervention of endurance training (Abkenar et al., 2019). Since an acute bout of endurance exercise did not induce any changes in the level of IL-1β and IL-18 in PBMCs, results show that moderate-intensity endurance training has the opposite effect than high-intensity endurance training, resulting in the decreased level of IL-18 and IL-1β (Abkenar et al., 2019). Moreover, Wagner et al. show that 6 weeks of intensive aerobic training at cycle ergometer at about 80% of VO_2max_ presents increased TNF-α level with a decreased level of BDNF (Wagner et al., 2015).

### Inflammatory Status After the Endurance Training in Parkinson's Disease

More interestingly, only a study of 12 PD subjects showed a decreased level of TNF-α after 8 weeks of moderate-intensity interval endurance training (Zoladz et al., 2014). A study of 14 people with PD after intensive endurance training showed no change in the inflammatory markers before and after the training (Landers et al., 2019). Both groups had a similar number of subjects and H&Y scale (2.3 ± 0.2 and, respectively, 2.0 ± 0.53), while in the Zoladz study, the subjects were older and had a longer disease duration (70.0 ± 3 years and 8.5 ± 1.3 years and, respectively, 63.5 ± 10.9 years and 4.92 ± 5.1 years). Different results might be caused by the different modes of training used in those studies. Zoladz et al. showed results after 8 weeks of moderate-intensity cycling program at 60–75% of HR_max_, while Landers showed results after 8 weeks of the treadmill run at 70-80% of HR_max_ (Zoladz et al., 2014; Landers et al., 2019). Thus, interval endurance training, even moderate-intensity endurance training, may have a more positive effect on the inflammatory response than high-intensity endurance training, not only in the group of people living with PD but also in healthy young adults (Zoladz et al., 2014; Abkenar et al., 2019; Landers et al., 2019). An interesting observation showed that TNF-α was decreased only in PD patients, while it increased in young, healthy subjects (Wagner et al., 2015). Landers et al. observed an increased level of IL-10 in both training groups, but in comparison to other studies, they used a statistically significant *p*-value of *p* < 0.1 rather than the more common *p* < 0.05 (Landers et al., 2019). An elevated level of IL-10 is desired, due to its anti-apoptotic role, especially in the context of neuroinflammation in the PD patient's brain. IL-10 is associated with anti-inflammatory activation of microglia in the brain and is responsible for silencing the inflammatory response against pathogens (Ogarra and Vieira, 2007). Moreover, by protecting IκB kinase, it inhibits the nuclear factor kappa-light-chain-enhancer of activated B cells (NF-κB) pathway, which leads to a decreased level of TNF-α (Petersen and Pedersen, 2005). Landers et al. observed that IL-10/TNF-α increases in both groups (low-intensity and high-intensity) after both modes of training were completed. Those findings might suggest that endurance training can enhance anti-inflammatory response in people with PD (Landers et al., 2019). Moreover, Lander et al. observed that TNF-α increases 6 months after the training completion in the group of high-intensity endurance training, while the level of these cytokines is the same before the intervention and after its completion. It increases in the time when subjects did not perform endurance training (Landers et al., 2019). In the control group (low-intensity group) increased level of TNF-α was also observed after 6 months of intervention, although it was not significant. Those findings may implicate that chronic endurance training may not decrease pro-inflammatory cytokines, but keeps its concentration on the same level, while after the training it is observed the progression of inflammation which could be previously stopped or slow down by the training (Landers et al., 2019). It can mean that endurance training may prevent or slow down the progression of inflammation in the context of PD. An increased level of IL-6 after an acute bout of endurance exercise has been reported in most of the studies, but no changes after the training program (Reihmane et al., 2013; de Gonzalo-Calvo et al., 2015; Wagner et al., 2015; Cabral-Santos et al., 2016; Brown et al., 2018). For many years, IL-6 has been reported to be a pro-inflammatory cytokine; however, research has demonstrated its important role in neuroprotection (Bauer et al., 2007). Its expression is mainly controlled by LPS, IL-1, and TNF-α (Lotz, 1993). As a neuroprotective interleukin, it increases the level of BDNF in the brain via A2aR (adenosine receptor two), which is involved in TrkB receptor transactivation (receptor for BDNF) and can modulate the effect of BDNF on transmission between synapses (Tebano et al., 2010; Perígolo-Vicente et al., 2014). This evidence may show that even though changes in the inflammatory status might not seem to be positive during physical exercise (due to elevated level of TNF-α and IL-6), this pro-inflammatory response may be involved in the increased level of beneficial BDNF.

### Improvement of the Inflammatory Status After a Bout of Endurance Exercise and Endurance Training

It is well-known that physical exercises provide many positive changes to our bodies. A lower BMI index and loss of fat tissue are some of the best-known aspects of being fit. It has been reported that most pro-inflammatory factors are produced by visceral fat tissue. A reduced level of visceral fat tissue leads to decreased levels of pro-inflammatory cytokines (Pedersen et al., 2003). Moreover, during skeletal muscle contraction, the expression of IL-6 increases, which may have a direct influence on the increased level of anti-inflammatory factors, such as IL-1 receptor antagonist and IL-10 and decreased levels of TNF-α and IL-1β (Pedersen et al., 2003; Starkie et al., 2003; Suzuki, 2019; Suzuki et al., 2020). Physical activity/training also modulates pathways involving nitric oxide (NO) and reactive oxygen species (ROS). During the first week of training or after intensive training, the level of pro-inflammatory factors and oxidative stress increases. This is a natural response to exercises (Scheele et al., 2009). Elevated levels of pro-inflammatory factors lead to elevated antioxidants levels (Brooks et al., 2008). With the training duration, ROS and NO levels decrease, while the level of antioxidants remains high. It has been reported that ROS participates in TNF-α catabolism in skeletal muscles (Blaser et al., 2016). Moreover, ROS and TNF-α produced as a result of physical exercises might be involved in the production of BDNF, which plays the main role in neuroprotection (Radak et al., 2016).

The most important factors in the context of neurodegenerative diseases such as PD, on which we focus in this review, are changes in the brain like increased level of BDNF or decreased level of pro-inflammatory cytokines such as TNF-α or IL-1β. Although systemic inflammation is present in PD patients and possible peripheral changes caused by intensive exercises would be desired, a positive response to exercise in the brain would be the most desirable effect. Looking for the Holy Grail in studies focused on the neurodegenerative diseases is dictated by finding a way to slow down the disease progression or to stop the further disease developments. Pathological changes in the neurodegenerative diseases are developing in the brain many years before the first symptoms occur (Postuma et al., 2012). Nowadays, discoveries of possible internal pathways or factors other than pharmacological treatment, that would be able to slow down the disease progression are the most desired. Research conducted using the animal model has shown that in the brain of rats after 6 weeks of interval and continuous exercises, H_2_O_2_ and TNF-α increase, respectively, with the increased levels of BDNF and glial cell line-derived neurotrophic factor (GDNF) (Afzalpour et al., 2015). Moreover, interval endurance training has a greater impact on all those factors. Conversely, another study has shown that 6 weeks of interval endurance training decrease the brain levels of IL-1β, TNF-α, IL-6, and IL-10 with the elevated level of BDNF and antioxidant defense mechanisms such as superoxide dismutase (SOD) and catalase (CAT) (Freitas et al., 2018). Both studies present a discrepancy in inflammatory marker results, but with an elevated BDNF level in both. More interestingly, Chennaoi et al. has shown that training decreases the level of IL-1β in the hippocampus and IL-6 in the cerebellum without any changes in the serum of rats (Chennaoi et al., 2008). These findings may indicate that physical intervention may not affect the peripheral level of cytokines or does not have a large influence on the peripheral immune system but directly affects the brain. The study focused on physical exercises in different postnatal stages of brain development that are present only in adolescent rats after interval endurance training, changes in levels of BDNF, TNF-α, and IL-10 but not IL-6 (Almeida et al., 2013). A serum inflammatory response to physical exercise was not detected in rats, but in human studies, this response is well-known (Petersen and Pedersen, 2005). Even though animal models are very helpful in studies examining changes in the brain after the physical exercise due to the lack of safe tools for use in humans, they may not be accurate.

### Increased Level of BDNF After Bout of Endurance Exercise and Endurance Training in Healthy Subjects

BDNF is one of the most important factors, playing a crucial role in learning, memory processes, as well as in neuronal survival (Howells et al., 2000). The increased levels in the brain might be the key to the positive effect of physical exercise. In the brain, BDNF is expressed in the cortex, hippocampus, midbrain, amygdala, hypothalamus, striatum, pons, and medulla oblongata, but the main source of BDNF is the dopaminergic neurons in the SNpc and striatum (Aid et al., 2007; Ventriglia et al., 2013). In the neuron, the highest levels of BDNF are in the cytosol as well as the dendrites (Zheng and Quirion, 2004; Zheng et al., 2012). Although BDNF plays an important role in neuron development (Scalzo et al., 2009), neuroplasticity, and memory processes (Andero et al., 2014), by activation of the antiapoptotic pathway and regulation of tyrosine hydroxylase (TH) and dopamine synthesis, it can regulate the process of dopamine-induced oxidative stress (Fukuchi et al., 2010). Moreover, activation of the TrkB/MAPK/ERK1/2/IP3K/Akt pathway regulates glutamate and NO neurotoxicity as well as damages caused by oxidative stress (Klintsova et al., 2004).

In this narrative review, we analyzed five articles with an acute bout of endurance exercise, seven studies with endurance training, and five articles with PD subjects in the context of changing the level of BDNF after the completion of the training program. In four studies BDNF increases after the single bout of endurance exercise completed by healthy, young men (Marquez et al., 2015; Cabral-Santos et al., 2016; Hwang et al., 2016; Slusher et al., 2018). Moreover, the increased level of BDNF was only temporal and decreased within the next 20-60 min (Marquez et al., 2015; Cabral-Santos et al., 2016; Hwang et al., 2016).

An acute bout of exercise shows an interesting phenomenon in which BDNF rapidly increases after the exercises but decreases in the short term after completion of the training (Marquez et al., 2015; Cabral-Santos et al., 2016; Hwang et al., 2016). Thus, the source of the peripheral BDNF after the exercise is unclear. Rasmussen et al. (2009) showed evidence that BDNF is released from the brain after physical exercise and can cross the BBB in both directions, as confirmed by Seifert et al. (2010). Thus, brain production may contribute almost 75% of the circulating BDNF, and it is said that ~25% might be released by other cells (Rasmussen et al., 2009). Knaepen et al. hypothesized that upregulated concentrations of circulating BDNF after physical exercise might be temporary (Knaepen et al., 2010). BDNF released into the bloodstream is quickly absorbed by other cells in the brain and/or by peripheral cells and can be used in further neuroprotective or neurotrophic processes. This hypothesis might confirm the findings reported by Matthews et al. and Pratesi et al. who showed that after the training and electric stimulation, BDNF levels increased in skeletal muscles, but it is not released into the bloodstream, suggesting that the increased level of BDNF might be secreted in the brain (Matthews et al., 2009; Pratesi, 2013). A similar suggestion was provided by Klinstova et al. in their research (Klintsova et al., 2004). During endurance training, many researchers have shown that the level of BDNF increases compared with the basal level in older adults (Erickson et al., 2011; Ruscheweyh et al., 2011; Leckie et al., 2014). Moreover, the basal level of this neurotrophin was higher before the last training session rather than at the beginning of the training cycle (O'Callaghan et al., 2019).

Seven studies with the program of endurance training on healthy people and its impact on the BDNF level were discussed in this review. Six studies out of seven were conducted on older adults and one with healthy young men (Erickson et al., 2011; Ruscheweyh et al., 2011; Voss et al., 2013; Leckie et al., 2014; Wagner et al., 2015; Maass et al., 2016; Schega et al., 2016). Results presented in this review are inconclusive. Three studies with moderate-intensity endurance training showed that serum BDNF level increases after the training program completion (Erickson et al., 2011; Ruscheweyh et al., 2011; Leckie et al., 2014) when four other studies show that the BDNF level does not change after the training program (Ruscheweyh et al., 2011; Voss et al., 2013; Maass et al., 2016; Schega et al., 2016). These discrepancies may be due to the intensity and volume of the training program, duration of hypoxia exposure, sports skills of study participants as well as study methodology and measurement techniques. When the age of all older participants is similar and might not play a significant role in the differences of this neurotrophin level, compared to the young adults it is seen that intensive training (about 77% of VO_2max_) leads to decreased BDNF level (Wagner et al., 2015).

The authors tried to explain why in their research presented opposite results than those in previous publications with older people by highlighting that the age might play a crucial role in the improvement of hippocampal volume and BDNF level (Wagner et al., 2015). Moreover, a similar study was conducted by Erikson et al. on the group healthy older people presenting that hippocampal volume and BDNF level increases after 7 weeks of moderate-intensity endurance training (Erickson et al., 2011). Both researchers highlighted that the age of participants might play a crucial role due to the brain with loss in volume in the older population might be more willing to increase its volume and cognitive function by increased BDNF, rather than brains of young people with more stable structures (Erickson et al., 2011; Wagner et al., 2015). Moreover, one meta-analysis presents that a training program has a lower size effect on the BDNF level in a healthy people group, rather than a single bout of exercise (Szuhany et al., 2015).

### Increased Level of BDNF After a Bout of Endurance Exercise and Endurance Training in People With Parkinson's Disease

In PD studies were observed an increased level of BDNF after moderate-intensity endurance training, high-intensity endurance training, and even after low-intensity training programs (Frazzitta et al., 2014; Zoladz et al., 2014; Marusiak et al., 2016; Landers et al., 2019; O'Callaghan et al., 2019). Moreover, one meta-analysis was conducted to analyze the influence of physical exercise on BDNF level in PD, showing that BDNF increases after the training program. Although, the authors highlighted that the data was provided only from two articles, which was the biggest limitation of this analysis (Hirsch et al., 2018). Only one study presented the effect of a single bout of endurance exercise on the BDNF level in PD, showing that an acute bout of endurance exercise does not affect the BDNF level in subjects with PD (O'Callaghan et al., 2019). We assume that maybe only repeated exercises for a longer time might induce BDNF transcription, not an acute intervention. Zoladz et al. presented that after 8 weeks of moderate-intensity interval endurance training, a higher level of BDNF was reported with a lower level of TNF-α and sVCAM-1 (Circulating Vascular Cell Adhesion Molecule-1) in the serum of PD patients (Zoladz et al., 2014). Moreover, the UPDRS (Unified Parkinson's Disease Rating Scale) decreased after the endurance training, showing that physical exercise might influence disease progression. More studies have shown that physical exercise intervention, especially interval endurance training or intensive rehabilitation, elevates the level of the BDNF in PD (Frazzitta et al., 2014; Landers et al., 2019; Malczynska et al., 2019; O'Callaghan et al., 2019). Two studies performed the measurements of the BDNF level not immediately after the last training session, but at the rest time, 48–72 h and 6–10 days after the training program completion (Marusiak et al., 2016; Landers et al., 2019). An elevated BDNF level at the rest time (not immediately after the training program) shows that changes in this protein level in the peripheral blood are still high after the end of the intervention (Marusiak et al., 2016; Landers et al., 2019). Although the BDNF level stays high after the training program, Landers et al. show that its level decreases below the basal level 6 months after the training intervention was completed. Those changes were accompanied by an increased level of TNF-α in the high-intensity training group, which can relate to the cessation of a positive training effect (Landers et al., 2019).

### Physical Activity and Factors Involved in the BDNF Upregulation

Although, the source of the increased BDNF in serum after physical exercise remains unclear, it has been demonstrated that after electric stimulation of the human skeletal muscle, elevated levels of BDNF mRNA are detected without any changes in its serum levels (Matthews et al., 2009; Pratesi, 2013). The process enhancing BDNF synthesis in the brain; however, remains incompletely understood, and possible hypotheses have been established to explain it. The first hypothesis states that intracellular Ca^2+^ is responsible for the increased BDNF level after physical activity (Fernandes et al., 2017). Ca^2+^ activates CaMKII (Ca^2+^/calmodulin-dependent protein kinase II), which activates the MAP-K (mitogen-activated protein kinase) pathway, leading in turn to cAMP Response Element-Binding Protein (CREB-binding protein) phosphorylation and CREB transcription activation. As the results of the pathway activation, the transcription of BDNF increases (Vaynman et al., 2004; Fernandes et al., 2017). Radak et al. formulated a hypothesis that involves ROS, assuming that as the effect of physical exercise, mitochondria produce more energy, which leads to elevated production of ROS (Radak et al., 2016). Increased production of ROS activates CREB-binding protein and further CREB and *Bdnf* transcription (Kim and Lee, 2013). Both hypotheses were created based on research related to moderate-intensity continuous training. *In vitro* research has shown that expression of BDNF and GDNF, another neurotrophic factor, can be regulated by elevated levels of H_2_O_2_, TNF-α, and increased activation of NADPH (Nicotinamide adenine dinucleotide phosphate) and its enhanced CREB phosphorylation (Kuno et al., 2006; Saha et al., 2006; Wang et al., 2006; Siamilis et al., 2008; Chan et al., 2010; Bałkowiec-Iskra et al., 2011). The role of TNF-α in the production of BDNF is not fully understandable, at least in the context of neurodegenerative disease. Miklic et al. showed that BDNF can be produced by astrocytes in response to stimuli (Miklič et al., 2004). Increased expression of BDNF in astrocytes by TNF-α can be induced through the NF-κB pathway (Saha et al., 2006). Moreover, it is known that TNF-α from the peripheral system can cross the BBB and activate promotor IV of *bdnf*, which leads to an elevated level of BDNF (Saha et al., 2006). In neurodegenerative disease, where TNF-α is upregulated, the BDNF level is decreased, which is why this hypothesis might be inaccurate in the context of neurodegeneration or diseases with chronic neuroinflammation (Sawada et al., 2006).

The TNF-α and ROS hypothesis might better fit the context of an acute bout of exercise or short training due to the increasing TNF-α level. A similar situation occurs during endurance training. Research has shown that after 6 weeks of high-intensity interval endurance training, BDNF, GDNF, TNF-α, and H_2_O_2_ increase in rats' brain; however, Freitas et al. showed that 6 weeks of high-intensity interval training increased BDNF, FRAP (Ferric-Reducing Antioxidant Power Assay), CAT, and SOD with decreased levels of pro-inflammatory cytokines such as TNF-α, IL-1β, IL-6, and IL-10 in the hippocampus of rats (Afzalpour et al., 2015; Freitas et al., 2018). Wu et al. showed that after LPS treatment and 4 weeks of training, the BDNF level increased in the brain of mice. The increased BDNF level was accompanied by an elevated level of its receptor TrkB (Wu et al., 2011). The authors highlighted that the BDNF level did not increase during the exercises until the mice received LPS treatment, demonstrating that the main cause of the BDNF elevation was inflammation caused by LPS. This study is important in the context of PD. By inducing inflammation, researchers were planning to assess the effect on the brain of PD patients with loss of dopaminergic neurons in SNpc. The results showed that the crucial factor in the positive effect of training on the brain in PD patients might be neuroinflammation (Wu et al., 2011). Experiments in animal models of PD have shown that long-term training causes an increased level of BDNF and TrkB receptor in the brain (Klintsova et al., 2004). Tuon et al. showed that after 8 weeks of endurance training in a rat model of PD, elevated levels of BDNF and TH were observed accompanied by decreased oxidative stress and alpha-synuclein levels (Tuon et al., 2012). Moreover, 10 weeks of treadmill endurance training in a mouse model of PD showed that the increased BDNF and GDNF could cause the preservation of dopaminergic neurons and protection from the inflammation caused by MPTP (1-methyl-4-phenyl-1,2,3,6-tetrahydropyridine) (Palasz et al., 2019). Through the signaling pathway activated by the TrkB receptor, BDNF regulates CREB phosphorylation, a transcription factor that is involved in the regulation of TH expression (Fukuchi et al., 2010). A lower level of TH has been observed in PD, leading to a lower level of dopamine (Feve, 2012). By BDNF stimulation, an elevated level of TH after training may lead to an elevated level of dopamine, which can slow down disease progression (Feve, 2012). In summary, animal models have shown that training affects the brain by leading to increased BDNF. In human studies, those changes are observed in the peripheral blood and might highlight the BDNF status in the brain (Klein et al., 2011). More research is needed to specify the mechanism responsible for the positive response to training in the brain. Due to the lack of safe techniques and methods allowing us to measure the level of BDNF in the human brain, so far, we must rely on animal models and imaging techniques like functional magnetic resonance and cognitive tests.

### Can miRNA Be Involved in the Regulation of Inflammatory Status During Endurance Training?

Past years have indicated that microRNA (miRNA) might play an important role in the regulation of gene expression. Researchers have shown that endurance training may regulate the expression of specific miRNAs. It was observed that the level of miRNAs changed in the plasma and serum after training, suggesting that miRNAs are released into the bloodstream during an exercise. Circulating miRNAs may play roles in cell communication and/or as mediators of gene expression in target cells (Kosaka et al., 2013). Many researchers have shown that different kinds of training lead to changes in different miRNA patterns. Most of them are highly expressed in muscle tissue and might be responsible for the regulation of processes related to muscle proliferation (Rooij et al., 2008). By studying many publications showing the changes in miRNA levels as a result of an acute bout of exercise and endurance training, we observed that some miRNAs are involved in the regulation of the apoptosis cycle and inflammatory factors. Moreover, increased levels of some miRNAs might be crucial in the prevention of PD due to the decreased levels of miRNAs in neurodegenerative disorders. The example miRNA involved in the regulation of inflammation is miR-146a, the expression of which is regulated by microbial components and pro-inflammatory factors. It acts via negative feedback as a regulator of the inflammatory response. miR-146a is responsible for expression regulation of tumor necrosis factor receptor-associated factor 6 (TRAF6), IL-6, and interleukin 1 receptor-associated kinase 1 (IRAK1) (Taganov et al., 2006; Iyer et al., 2012). Moreover, it is a regulator of the monocyte pro-inflammatory response and negatively regulates the pro-inflammatory pathway via NF-κB (Taganov et al., 2006; Iyer et al., 2012). Upregulated levels of miR-146a have been reported in response to acute endurance training (Baggish et al., 2011, 2014; Nielsen et al., 2014; Wardle et al., 2015). A decreased serum level of miR-146a has been observed in PD patients (Ma et al., 2016; Caggiu et al., 2018). It is still unclear whether its level is correlated with the increased level of TNF-α or other inflammatory markers. Other miRNAs like miR-222 and miR-221, involved in the apoptosis and progression of PD, have been reported to increase after prolonged training (Baggish et al., 2011; Lupini et al., 2013; Wardle et al., 2015; Ma et al., 2016; Schmitz et al., 2018). Upregulation of specific miRNAs in PD following physical exercises might introduce a positive effect on pathological biochemical changes in the brain of PD patients. Unfortunately, there is no strong evidence to date that the previously reported miRNA regulators of processes associated with apoptosis and inflammation might have a positive effect on the biochemical changes after the training. No data has supported this effect in PD patients, and publications with healthy young subjects do not show a correlation between specific inflammatory markers and the miRNAs they regulate. Confirmation of this hypothesis might highlight molecular processes underlying the basis of the positive effect of the endurance training. Thus far, we can only speculate that they may affect other cells in the peripheral system as well as the CNS by changes in their bloodstream levels.

## Conclusions and Practical Applications

In this narrative review, we innovatively discuss changes in the inflammatory status and BDNF level caused by acute physical exercise and endurance training. The past 10 years brought a new highlight on the positive effect of performing training programs. In general, it appears to have a better influence on the organism by improving the condition of the body, regulation of glucose intake, heart rate, body mass, and mood. The following findings were discussed in this narrative review:

– An acute bout of endurance exercise temporarily improves the inflammatory status by increasing the levels of cytokines such as IL-10, IL-6, and TNF-α, accompanied by a temporary improvement in the BDNF level.
– A different inflammatory status pattern was observed in measurements after the endurance training program in healthy young adults.
– In PD studies, TNF-α decreases or stays at the same level after the endurance training program. IL-10 elevates after high-intensity endurance training and after low-intensity training.
– After the end of the physical intervention the level of pro-apoptotic cytokines might elevate in people with PD, what can be associated with the disease progression.
– BDNF level increases after a single bout of endurance exercise in healthy young people but drastically decreases within the next 60 min.
– No changes in the BDNF level after an acute bout of exercise was observed in PD participants.
– Results of the influence of endurance training on the BDNF level in older people are inconclusive due to many studies with no significant changes in BDNF level and studies with elevated neurotrophic factor.
– Only one study discussed in this narrative review presented the BDNF level after an intensive endurance training program in young people. The concentration of this protein was decreased compared to its basal level.
– In PD studies, BDNF level elevates after the last endurance exercise session of the training program and at the rest time, 2–10 days after the training completion. After the intervention, the BDNF level might stay high to decrease within months after the end of the training program.

Most studies have shown that the BDNF level increases in the serum of older adults and PD patients. It is assumed that the intensity of the endurance training may also have an impact on the changes in inflammatory status and the BDNF level. To date, the data indicate that BDNF might lead to a decreased level of inflammatory factors, and its level might be improved by increased levels of ROS, TNF-α, and IL-6 after the endurance exercises. This phenomenon can be considered to be a neuroprotective mechanism leading to protection from the negative effects of inflammation caused by physical exercises. Data show that responses from the immune system and brain are different in studies with healthy people and studies with older adults and people living with PD. Due to the age, health status, intervention time, and intensity, it should not be assumed that changes induced by endurance training or an acute bout of endurance exercise might be the same for all healthy people or people who suffer from a chronic disease, not only neurological. Using this knowledge in the context of PD, we assume that endurance training might provide positive effects as support to pharmacotherapy due to its positive effect on cognitive function by the improvement of the BDNF level and increasing the number of anti-inflammatory cytokines. It is important to mention that even though an endurance training program might not induce any significant changes in the group of PD participants, it still might have a positive effect on disease progression by stopping the increases in the inflammatory status. So far, data indicate that moderate or high-intensity endurance training might bring many benefits such as an improvement of cognitive function and slow down the progression of systemic inflammation rather than only levodopa supplementation. Performing regular physical activity, not only as an intensive endurance training program, but also as everyday activities such as walking, stretching, cycling outside on a bicycle, and other low-intensity exercises might bring a positive effect for people living with PD and older healthy people. It should be considered to encourage people living with PD and older people to conduct daily activities and perform intensive endurance program in the way it is proper for their health status and preferences.

## Data Availability Statement

The original contributions presented in the study are included in the article/supplementary material, further inquiries can be directed to the corresponding author/s.

## Author Contributions

PM-S, MC, and AS conceptualization, writing, and editing. CM and SA supervised work. All authors have read and approved the final version of the manuscript and made a significant contribution to this study.

## Conflict of Interest

The authors declare that the research was conducted in the absence of any commercial or financial relationships that could be construed as a potential conflict of interest.
